# Is smoking heaviness causally associated with alcohol use? A Mendelian randomization study in four European cohorts

**DOI:** 10.1093/ije/dyy027

**Published:** 2018-03-02

**Authors:** Michelle Taylor, Line Rode, Johan Bjørngaard, Amy E Taylor, Stig E Bojesen, Bjørn O Åsvold, Maiken E Gabrielsen, Glyn Lewis, Børge G Nordestgaard, Pål R Romundstad, Matthew Hickman, Marcus R Munafò

**Affiliations:** 1 Population Health Sciences, Bristol Medical School, University of Bristol, Bristol, UK; 2 MRC Integrative Epidemiology Unit (IEU), University of Bristol, Bristol, UK; 3 UK Centre for Tobacco and Alcohol Studies, School of Experimental Psychology, University of Bristol, Bristol, UK; 4 Department of Clinical Biochemistry and the Copenhagen General Population Study, Copenhagen University Hospital, Herlev and Gentofte Hospital, Herlev, Denmark; 5 Department of Public Health and Nursing, NTNU, Norwegian University of Science and Technology, Trondheim, Norway; 6 Forensic Department and Research Centre Brøset St Olav’s University Hospital Trondheim, Trondheim, Norway; 7 Faculty of Health and Medical Sciences, University of Copenhagen, Copenhagen, Denmark; 8 Department of Endocrinology, St Olavs Hospital, Trondheim University Hospital, Trondheim, Norway; 9 KG Jebsen Center for Genetic Epidemiology, Department of Public Health and Nursing, NTNU, Norwegian University of Science and Technology, Trondheim, Norway; 10 Department of Laboratory Medicine, Children’s and Women’s Health, NTNU, Norwegian University of Science and Technology, Trondheim, Norway; 11 Division of Psychiatry, University College London, London, UK

**Keywords:** Mendelian randomization, licit drugs, ALSPAC, HUNT, CGPS, UK Biobank

## Abstract

**Background:**

Observational studies have shown that tobacco and alcohol use co-occur, but it is not clear whether this relationship is causal.

**Methods:**

Using data from the Avon Longitudinal Study of Parents and Children (ALSPAC) and UK Biobank, we used observational methods to test the hypothesis that smoking heaviness increases alcohol consumption. Mendelian randomization (MR) analyses were then used to test the causal relationship between smoking heaviness and alcohol consumption using 55 967 smokers from four European studies [ALSPAC, The Nord-Trøndelag Health Study (HUNT), the Copenhagen General Population Study (CGPS) and UK Biobank]. MR analyses used rs1051730/rs16969968 as a genetic proxy for smoking heaviness.

**Results:**

Observational results provided evidence of an association between cigarettes per day and weekly alcohol consumption (increase in units of alcohol per additional cigarette smoked per day = 0.10, 95% confidence interval (CI) 0.05 to 0.15, *P* ≤ 0.001 in ALSPAC; and 0.48, 95% CI 0.45 to 0.52, *P* ≤ 0.001 in UK Biobank). However, there was little evidence for an association between rs1051730/rs16969968 and units of alcohol consumed per week across ALSPAC, HUNT, CGPS and UK Biobank (standard deviation increase in units of alcohol per additional copy of the risk allele = –0.004, 95% CI –0.023 to 0.016, *P*=0.708, *I*^2^ = 51.9%). We had 99% and 88% power to detect a change of 0.03 and 0.02 standard deviation units of alcohol per additional copy of the risk allele, respectively.

**Conclusions:**

Previously reported associations between smoking and alcohol are unlikely to be causal, and may be the result of confounding and/or reverse causation. This has implications for public health research and intervention research.

**Key Messages**

- Observational studies have shown consistent strong evidence for an association between tobacco and alcohol use.
- It has been suggested that reductions in tobacco use can be used as an intervention target for alcohol.
- Our study suggests that heaviness of smoking does not causally influence level of alcohol consumption.
- It is likely that previous findings were the subject of confounding, reverse causation or bias.
- These findings could have implications for targeting smoking behaviour in interventions.

## Introduction

Smoking and alcohol are among the most important preventable causes of morbidity and mortality.^1–4^ Many studies have examined the relationship between tobacco and alcohol use, with several of these focusing on smoking as a risk factor for later alcohol use in both adolescence^5–11^ and adulthood.^12–16^ However, current evidence is inconsistent and the observational data used in such studies are difficult to interpret due to the potential for unmeasured confounding and reverse causation. Determining whether there is a causal association between tobacco and alcohol use is important in the attempt to reduce the use of these drugs, as one could use prevention of tobacco use as a means of reducing alcohol misuse.

Mendelian randomization (MR) uses genetic variants known to be associated with an exposure of interest as a method of testing whether there is a causal association between exposure and disease.^17^ MR is based on three assumptions. First, the genetic instrument being used must be associated with the exposure of interest. Second, the genetic instrument must only influence the outcome through the exposure of interest. Third, the genetic instrument cannot be associated with any factors that confound the relationship between the exposure and the outcome.^17^ If the assumptions of MR hold, genetic variants associated with an exposure of interest should be independent of confounding factors.^18^ Furthermore, as genotype is determined at conception, it cannot be influenced by any stage of the disease process and therefore estimates cannot be the result of reverse causation.^17^^,^^18^

We hypothesized that a genetic determinant of smoking phenotypes would be associated with increased levels of alcohol use and would therefore provide evidence of a causal relationship (MR). To test this, we first assessed the relationship between heaviness of smoking and weekly alcohol consumption using observational methods. Data from the Avon Longitudinal Study of Parents and Children (ALSPAC) and UK Biobank were used. In MR analyses, we used two single-nucleotide polymorphisms (SNPs) in the nicotine acetylcholine receptor gene cluster (*CHRNA5-CHRNA3-CHRNB4*): rs1051730 and rs16969968, which are highly correlated and therefore can be used interchangeably.^19^ The rs16969968 SNP is a missense mutation that codes for a change in amino acid from aspartate to asparagine in the α5 nAChR subunit protein and is therefore of functional significance. Conversely, rs1051730 is a coding synonymous variant and is more likely to act as a proxy for a functional SNP.^20^ These variants have been shown to be robustly associated with the number of cigarettes consumed per day^21–26^ and have previously been used in MR studies.^27–35^ MR analyses were carried out using four European cohorts [ALSPAC, the Nord-Trøndelag Health Study (HUNT), the Copenhagen General Population Study (CGPS) and UK Biobank] with results from all studies being meta-analysed.

## Methods

### Study populations

Four European cohort studies were utilized in this analysis (ALSPAC, HUNT, CGPS and UK Biobank). ALSPAC is a longitudinal birth cohort study situated in south-west England that recruited more than 14 000 pregnant women between 1991 and 1992.^36^^,^^37^ HUNT invited individuals in Nord-Trøndelag County in Norway who were aged 20 years or older between 1995 and 1997 to take part in the second wave of the study, and successfully recruited 65 215 individuals.^38^^,^^39^ CGPS is a study comprising randomly selected Copenhagen residents aged 20–100 years.^27^ UK Biobank recruited over 500 000 men and women (aged 37–73 years) between 2006 and 2010.^40^ Full description of each of the cohorts can be found in Supplementary Methods, available as Supplementary Data at *IJE* online.

### Phenotypic measures

#### Smoking

Cigarettes per day in individuals who reported smoking was a continuous variable—a measure that has previously shown association with rs16969968/rs1051730.^23^^,^^41^^,^^42^ In ALSPAC, these data were collected during pregnancy (18 weeks’ gestation), but addressed regular smoking status pre*-*pregnancy. In UK Biobank, participants were asked about current and past smoking status during the baseline computer-administered questionnaire. Individuals were classed as current, former or never smokers. Individuals reporting regular use of pipes or cigars were excluded from analyses.

#### Alcohol

Alcohol consumption was a continuous measure of units of alcohol consumed per week, derived from the reporting of frequency of alcohol consumption. Different questions were asked in each of the four cohorts but all allowed the calculation of average weekly intake in units. Further information on the derivation of this variable is provided in Supplementary Table 1, available as Supplementary Data at *IJE* online. Non-drinkers were excluding from analyses.

### Confounders

Potential confounders for the observational analysis (conducted in ALSPAC and UK Biobank; see ‘Statistical analysis’ below) included: sex (UK Biobank only), age in years, social class (0 ‘*III manual skilled, IV and V unskilled manual or casual workers or those who rely on state for their income*’ and 1 ‘*I and II professional occupations and managerial and technical occupations and III non-manual skilled workers*’^43^ (ALSPAC only); highest level of education (0 ‘*college degree or higher*’ and 1 ‘*A level equivalent or lower*’) (UK Biobank only); partner’s smoking (reported by the mother) (0 ‘*no*’ and 1 ‘*yes*’) (ALSPAC only); partner’s drinking (reported by the mother) (0 ‘*never/very occasionally*’, 1 ‘*occasionally*’ and 2 ‘*daily*’) (ALSPAC only).

### Genetic measures

Genotyping information for ALSPAC, HUNT, CGPS and UK Biobank is provided in Supplementary Methods, available as Supplementary Data at *IJE* online. The rs1051730/rs16969968 variants were used as a proxy for heaviness of smoking (measured here as cigarettes per day) and were coded 0, 1 and 2 for genotypes *CC*, *CT* and *TT*, respectively. The minor allele frequency (MAF) was 0.33 for both rs1051730 and rs16969968.

### Statistical analysis

Linear regression was used to assess the association between cigarettes per day and units of alcohol per week [adjusted for age (both cohorts), social class, partner’s smoking and drinking (ALSPAC only), sex and education (UK Biobank only)] in ALSPAC and UK Biobank only.

Using data from ALSPAC and UK Biobank, we tested the association between rs1051730/rs16969968 and cigarettes per day as a test of the first assumption of MR (genotype must be associated with the exposure). We examined associations between rs1051730/rs16969968 and confounders to test the third assumption of MR (that the genetic instrument cannot be associated with any factors that confound the relationship between the exposure and the outcome). Both CGPS^27^ and HUNT^28^ have previously published the association between rs16969968/rs1051730 and units of alcohol per week. The aim of these previous analyses was not to assess the relationship between smoking heaviness and alcohol consumption; however, both tested the association between rs1051730 and alcohol consumption as a test for genetic pleiotropy (i.e. considering alcohol as a potential confounder in their analysis). Additionally, these previous analysis have assessed the association between rs1051730 and smoking behaviour and a range of relevant confounders.^27^^,^^28^

Deviation from Hardy-Weinberg equilibrium was assessed as a test of missingness that might arise from genotyping errors, clinical ascertainment or by chance.^44^

In each of the four cohorts (ALSPAC, HUNT, CGPS and UK Biobank), units of alcohol per week (excluding non-drinkers) (Supplementary Table 1, available as Supplementary Data at *IJE* online) was standardized (i.e. converted to a Z-score) for consistency between datasets. We then tested for association between rs1051730/rs16969968 and standardized units per week stratified by smoking status (current smokers/former smokers/never smokers). Analysis in never and former smokers tests the pleiotropy assumption of MR (i.e. the genotype cannot be associated with the outcome through any other phenotype). Additional sensitivity analysis was conducted stratifying the sample in ever smokers and never smokers.

We opted to use linear regression over two-stage least-squares regression. This is because the second assumption of MR (that the SNP should only be associated with the outcome through the exposure of interest) is likely to be violated when the phenotype (e.g. cigarettes per day) does not adequately capture the exposure through which the genetic variant operates. This has been described in detail elsewhere.^45^^,^^46^ In brief, results from two-stage least-squares regression may be biased when this assumption is violated. In this situation, the genetic variant is still a valid instrument to provide evidence of causality, but is not a valid instrument for quantifying the effect of the measured phenotype on the outcome. We assume a constant effect of smoking on alcohol consumption and, as a result, we identify the average effect of smoking heaviness on alcohol consumption in the sample.

The effect sizes between rs16969968/rs1051730 and standardized units per week stratified by smoking status for each study were pooled in a meta-analysis. We used DerSimonian and Laird random-effects meta-analysis^47^ using the *metan* command in Stata 13.^48^^,^^49^

Quanto^50^ was used to calculate the sample size required to obtain different effects in the MR analyses. A continuous trait design was specified with a gene-only hypothesis, using a desired power of 0.80, a significance level of 0.05 (two-sided) and a log additive mode of inheritance.

All analysis was carried out following STROBE (Strengthening the Reporting of Observational Studies in Epidemiology) guidelines (Supplementary Table 2, available as Supplementary Data at *IJE* online). The Instrumental Variable Checklist^51^ (Supplementary Table 3, available as Supplementary Data at *IJE* online) was used for MR analysis and the instrumental variable flow chart considered throughout analyses and reporting.^52^

## Results

### Observational results

A total of 8030 individuals had genetic information collected in ALSPAC and provided information on their smoking status. Of these, 2198 (27.4%) were smokers and provided information on alcohol consumption. The median number of cigarettes per day and units of alcohol per week were 15 (IQR = 15) and 4 (IQR = 3.5), respectively. Following inclusion of covariate data, the sample size for the complete case analysis was 1359 (Supplementary Table 4, available as Supplementary Data at *IJE* online). In UK Biobank, 335 921 individuals had genetic information and provided data on their smoking status. Of these, 30 241 (9.0%) were smokers and provided information on alcohol consumption. The median number of cigarettes per day and units of alcohol per week were 15 (IQR = 10) and 8 (IQR=14), respectively. Following inclusion of covariate data, the sample size for the complete case analysis was 15 323 (Supplementary Table 5, available as Supplementary Data at *IJE* online).

There was strong evidence for an association between cigarettes consumed per day and units of alcohol per week [change in units per week for each additional cigarette per day smoked = 0.09, 95% confidence interval (CI) 0.04 to 0.15, *P* ≤ 0.001 in ALSPAC; and 0.65, 95% CI 0.61 to 0.69, *P* ≤ 0.001 in UK Biobank], which remained after adjustment for confounders (change in units per week for each additional cigarette smoked = 0.10, 95% CI 0.05 to 0.15, *P* ≤ 0.001 in ALSPAC; and 0.48, 95% CI 0.45 to 0.52, *P* ≤ 0.001 in UK Biobank) (Table 1).

**Table 1:** Unadjusted and adjusted effect sizes for observational analysis examining the association between cigarettes per day and units of alcohol per week in ALSPAC and UK Biobank

Adjustment	*N*	Coef*	95% CI	LR(χ^2^)	LR test *P*-value
**ALSPAC**					
Unadjusted (all available data)	2198	0.11	0.07–0.16	23.02	≤0.001
Unadjusted (complete case analysis)	1359	0.09	0.04–0.15	12.01	≤0.001
Socio-economic position	1359	0.10	0.04–0.15	12.94	≤0.001
Age	1359	0.09	0.04–0.15	12.39	≤0.001
Partner’s smoking	1359	0.09	0.04–0.15	12.10	≤0.001
Partner’s drinking	1359	0.10	0.05–0.15	13.67	≤0.001
Fully Adjusted	1359	0.10	0.05–0.15	14.66	≤0.001
**UK Biobank**					
Unadjusted (all available data)	15 462	0.65	0.61–0.69	1090.35	≤0.001
Unadjusted (complete case analysis)	15 323	0.65	0.61–0.69	1067.82	≤0.001
Education	15 323	0.65	0.61–0.69	1037.70	≤0.001
Age	15 323	0.65	0.61–0.69	1047.03	≤0.001
Sex	15 323	0.48	0.44–0.51	611.50	≤0.001
Fully adjusted	15 323	0.48	0.45–0.52	624.42	≤0.001

* Coefficients describe the increase in units of alcohol per week for each additional cigarette smoked per day. LR, likelihood ratio.

### MR results

In both ALSPAC and UK Biobank, there was evidence for an association between rs1051730/rs16969968 and cigarettes per day in those who smoked (change in cigarettes smoked per day for each additional copy of the risk allele = 0.91, 95% CI 0.41 to 1.40, *P* ≤ 0.001 in ALSPAC; and 0.95, 95% CI 0.79 to 1.12, *P* ≤ 0.001 in UK Biobank), which is consistent with previous evidence that each additional copy of the risk allele is responsible for an approximately one-cigarette-per-day increase in smoking heaviness.^25^^,^^26^ There was no evidence for association between rs1051730/rs16969968 and potential confounding factors in ALSPAC or UK Biobank (Supplementary Tables 4 and 5, available as Supplementary Data at *IJE* online) or a departure from Hardy-Weinberg equilibrium (ALSPAC χ^2^*P*-value = 0.34; HUNT χ^2^*P*-value = 0.12; CGPS χ^2^*P*-value = 0.32; UK Biobank χ^2^*P*-value = 0.89).

**Figure 1:**
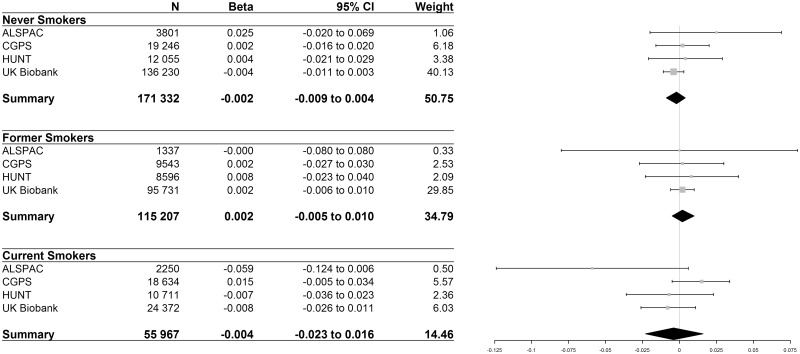
Effect sizes represent the standard deviation increase in units of alcohol per week for each additional copy of the minor (risk) allele. P values for association in: never smokers = 0.496, former smokers = 0.549, current smokers = 0.708. Test of heterogeneity: never smokers I2 = 0.0%, p = 0.558; former smokers I2=0.0%, p = 0.986; current smokers I2 = 51.9%, p= 0.101. Note: weights are from random effects meta-analysis.

In total, 55 967 smokers were included when pooling results from the ALSPAC, HUNT, CGPS and UK Biobank studies. There was little evidence for an association between rs1051730/rs16969968 and units of alcohol per week (where β coefficients represent the standard deviation change for each additional copy of the risk allele) in never (β = –0.002, 95% CI –0.009 to 0.004, *P* = 0.49), former (β = 0.002, 95% CI –0.005 to 0.010, *P* = 0.55) or current smokers (β = –0.004, 95% CI –0.023 to 0.016, *P* = 0.71) (Figure 1). Additionally, there was little evidence for an association between rs16969968/rs1051730 and units of alcohol per week in ever smokers (β = 0.003, 95% CI –0.004to 0.009, *P* = 0.45) (Supplementary Figure 1, available as Supplementary Data at *IJE* online). In ALSPAC and UK Biobank, MR results using all available data were consistent with those using complete case data from the observational analysis (Supplementary Table 6, available as Supplementary Data at *IJE* online).

We calculated that our sample size of 55 967 current smokers had at least 99% and 88% power to detect a change of 0.03 and 0.02 standard deviations of units of alcohol per additional copy of the minor allele, respectively.

## Discussion

We corroborate the strong association between tobacco and alcohol consumption in observational data sets using ALSPAC and UK Biobank cohorts. However, we found no clear evidence of a causal effect when using a genetic marker of smoking heaviness in an MR framework using current smokers from four European cohorts. Our analysis suggests that the reported associations between smoking and alcohol could be strongly influenced by confounding factors and/or reverse causality, and that smoking heaviness is not causally associated with alcohol consumption.

Two studies have assessed the relationship between tobacco and alcohol using MR. The results reported in this manuscript are in agreement with Vrieze and colleagues, who did not report an association.^53^ However, as the previous analysis uses an adolescent sample, the results may not be comparable with an adult sample, such as those we report here. Vink and colleagues reported that both cigarettes per day and smoking cessation were associated with glasses of alcohol per week when using polygenic risk scores as proxies for smoking phenotypes.^54^ This analysis used weak *P*-value thresholds to generate their polygenic risk scores and therefore provide evidence for shared aggregated genetic risk factors between tobacco and alcohol use, rather than testing for a causal relationship. Following meta-analysis of data from four studies, the sample size of the analysis we report here increased to 55 967 and is therefore much larger than that reported by Vink and colleagues, providing more robust evidence for no causal association between heaviness of smoking and alcohol consumption.

As alcohol consumption does not remain stable over time, smoking may have a causal effect on levels of alcohol consumption regardless of when people start drinking. Here, we have examined the dose—response relationship between heaviness of smoking among current smokers with levels of alcohol consumption, not the effect of smoking initiation on subsequent alcohol use. By examining this using MR, our results cannot be influenced by reverse causation. One study conducted by Irons and colleagues (2007) examined the causal effect of alcohol use on tobacco use using MR finding little evidence for an effect.^55^ This study, in conjunction with the results reported here, provides evidence that previously reported associations between tobacco and alcohol use are not the result of reverse causation and that confounding factors are responsible for the co-occurrence of these substances.

A number of limitations need to be considered when interpreting these results. First, when excluding women with missing data on all genetic, outcome, exposure and covariate measures, there is a large amount of attrition from the original ALSPAC dataset. Second, both smoking and alcohol consumption in this study were assessed using self-report. The potential effect of this limitation is reduced by the use of a genetic variant as a determinant of smoking, as it is unlikely to vary with regard to reporting bias. Nevertheless, biological assessment at least for tobacco use (based on cotinine data for smoking) would be advantageous in any further studies.^56^ However, alcohol biomarkers for chronic consumption are unreliable.^57^ Third, it is likely that MR analysis is underpowered to rule out any association between tobacco and alcohol. We would not expect to see the same effect size between the non-genetic observational analysis and the MR analysis (since the genetic variants only explain a small proportion of the variance in heaviness of smoking). However, as the observed effects are very close to the null, the conclusion can be made that the MR result is consistent with a null result. Furthermore, the meta-analysis of results with those from the HUNT and CGPS cohorts provide further evidence that is consistent with the null hypothesis. Fourth, the design of MR and meta-analysis are susceptible to population stratification. There is potential for population stratification between each of the datasets used. However, the effect in each of these studies has been examined separately and no difference was observed, suggesting it is reasonable to conclude that population stratification is not affecting these results. We did observe heterogeneity between the observational results in ALSPAC and UK Biobank, but the direction of the association was consistent. The differences in the magnitude of the association could be explained by differences in age and gender between the two populations, the fact that the data were collected ∼15 years apart or that the ALSPAC sample comprised individuals who might have been trying to get pregnant at the time. Finally, we cannot completely rule out the possibility that collider bias may affect these results. As the genetic variant influences likelihood of smoking cessation,^58^ stratifying analyses into former and current smokers could induce collider bias. However, there is little evidence to suggest that the variant influences smoking initiation, so stratification of results into ever and never smokers should be less problematic. Furthermore, collider bias could also arise if selection into the study samples is related to both alcohol consumption and rs16969968/rs1051730 (if heavier smoking makes individuals less likely to participate). This is most likely to be an issue in UK Biobank, which has very low participation rates and is not likely to be very representative of individuals of the target age group living in the UK.^59^ When excluding UK Biobank from the meta-analysis, results remained the same (Supplementary Figure 2, available as Supplementary Data at *IJE* online).

MR techniques are particularly pertinent to behavioural exposures—such as tobacco smoking, alcohol consumption, cannabis and other drug use—which cannot be randomized, cluster with other risk behaviours and confounders, and lack effective interventions that could be used in trials that randomize the removal of the exposure. We find no clear evidence for the prevailing assumption that tobacco causally affects alcohol consumption, which has great implications for public health and intervention research. Interventions that target reductions in tobacco consumption may not necessarily also lead to any change in alcohol consumption, and interventions that seek to target both smoking and alcohol will need to incorporate active ingredients for each substance.

## Supplementary Material

Supplementary Data
