# Supplementary material for: Is smoking heaviness causally associated with alcohol use? A Mendelian randomization study in four European cohorts
Source: Int J Epidemiol. 2018 Mar 2;47(4):1098–105. doi: 10.1093/ije/dyy027 (PMC6124618; doi:10.1093/ije/dyy027)
Supplement: Supplementary Data [file dyy027_supplementary_methods.docx]

**Supplementary Methods: Cohort descriptions and genotypic information**

***Avon Longitudinal Study of Parents and Children (ALPSAC)***

ALSPAC is a longitudinal study situated in South West England that recruited more than 14 000 pregnant women between 1991 and 1992. Comparison with the 1991 census shows the sample was broadly representative of the British population (1). Ethics approval was obtained from the ALSPAC Ethics and Law Committee and the Local Research Ethics Committees. Further information of the recruitment process is available elsewhere (1, 2). The study website contains details of all data through a searchable data dictionary (3).

The cohort description for ALSPAC has been provided in the main text. Genotypic information was available from 10 015 of the ALSPAC mothers. Centre National de Génotypage (CNG) carried out DNA genotyping on the Illumina human600W-quad array and genotypes were called with Illumina GenomeStudio. PLINK (v. 1.07) (4) was used to carry out quality control measures on each of the subjects and 557 124 genotypes SNPs, details of which are available elsewhere (5). A total of 8340 subjects and 526 688 SNPs passed these quality control filters. Autosomal SNPs were imputed against the HapMap CEU population (release 22) using MaCH (v1.0.16) and NCBI build 36, HapMap 3 release 2.

***Copenhagen General Population Study (CGPS)***

CGPS was started in 2003 and is still recruiting. Randomly selected Copenhagen residents aged 20 to 100 years were invited to complete an interviewer-administered questionnaire, undergo a physical examination and provide a blood sample (6). The study was conducted according to the Declaration of Helsinki. All participants gave written informed consent, and Herlev Hospital and a Danish scientific ethics committee approved the study (H-KF01-144/01). DNA was extracted from blood samples using the ABI PRISM 7900HT Sequence Detection System to genotype rs1051730 using a TaqMan assay. The genotype was in Hardy-Weinberg equilibrium (χ^2^ P-value = 0.32) (6). Derivation of the units of alcohol per week measure is provided in **Supplementary Table 2**.

***Nord-Trøndelag Health Study (HUNT)***

Individuals in Nord-Trøndelag County in Norway who were aged 20 years or older were invited to participate in the second wave of HUNT Study between 1995 and 1997. Of the 93 898 individuals invited, 65 215 (69%) participated in the study which has been described in detail elsewhere (7, 8). Briefly, participants completed a self-administered questionnaire that covered a wide range of health topics and attended a clinic session where clinical measures were collected and a non-fasting blood sample was drawn. The population is predominantly (>97%) Caucasian (7). DNA was extracted from blood samples and stored at HUNT Biobank, Levanger. The rs1051730 variant was genotyped at HUNT Biobank using a TaqMan assay (Assay ID: C_9510307_20, Applied Biosystems) on an Applied Biosystems 7900HT Fast Real-Time PCR System, as previously described (9). The call rate cut-off was set to 90%. In total, DNA was available for 57 082 (87.5%) and rs1051730 was successfully genotyped in 56 664 (99.3%) participants. The genotype was in Hardy Weinberg equilibrium (χ^2^ P-value = 0.115). Derivation of the units of alcohol per week measure is provided in **Supplementary Table 2**.

***UK Biobank***

The UK Biobank ([www.ukbiobank.ac.uk](http://www.ukbiobank.ac.uk)) recruited over 500,000 men and women (aged 37 to 73 years) between 2006 and 2010 (10). Participants attended one of the 21 assessment centres in England, Wales and Scotland, where they provided information on demographic, lifestyle factors and medical history through interviews and questionnaires and had physical measurements and blood, urine and saliva samples taken. The full protocol for the study is available online: www.ukbiobank.ac.uk/wp-content/uploads/2011/11/UK-Biobank-Protocol.pdf. The UK Biobank study was approved by the North West Multi-Centre Research Ethics Committee and all participants provided written informed consent to participate in the UK Biobank study.

DNA was extracted from blood samples using the Promega Maxwell 16 Blood DNA Purification Kit (AS1010), which uses magnetic bead technology to purify the DNA. Genetic data was available on 488,377 UK Biobank participants and were genotyped using either the Affymetrix UK BiLEVE Axiom array or the Affymetrix UK Biobank Axiom array. These two arrays are very similar, with an overlap of around 95%. Further information on the genotyping process has been provided elsewhere (11). Information on in house filtering steps has been documented elsewhere (12) and resulted in 337 109 individuals remaining in the analysis. The smoking heaviness related SNP rs16969968 did not show clear evidence for deviation from Hardy Weinberg equilibrium (χ^2^ P-value = 0.89).

Participants were asked about current and past tobacco (cigarette, pipe, cigar or other) smoking behaviour in a computerized questionnaire. A full list of the questions is available at: <http://biobank.ctsu.ox.ac.uk/crystal/docs/TouchscreenQuestionsMainFinal.pdf>. The following questions were asked about current and past smoking status: “Do you smoke tobacco now?” (Yes, on most or all days, Only occasionally, No, Prefer not to answer) and “In the past, how often have you smoked tobacco?” (Smoked on most or all days, Smoked occasionally, Just tried once or twice, I have never smoked, Prefer not to answer). Individuals who indicated that they had tried tobacco but were not past or current daily or near daily smokers were also asked “Have you smoked more than 100 cigarettes in your lifetime?”. From these questions, the following smoking status categories were defined: never smokers (individuals who had smoked less than 100 cigarettes in a lifetime), former smokers (who had smoked more than 100 cigarettes but were not current smokers) and current smokers. Where individuals did not know or did not say whether they had consumed more than 100 cigarettes in their lifetime, those who said they had tried one or two in the past were classified as never smokers and those who said they smoked occasionally in the past as former smokers). Participants were also asked the question “About how many cigarettes do you smoking on average each day?” and provided a number which was used to determine the cigarettes per day smoking by each individual.

Information about alcohol consumption was collected in the same computerised questionnaire. Individuals were asked about the frequency of their current alcohol consumption (Daily or almost daily/ Three or four times a week/ Once or twice a week/ One to three times a month/ Special occasions only/ Never). Individuals reporting at least weekly drinking were asked how many measures of red wine (glasses)/white wine or champagne (glasses)/beer or cider(pints)/spirits(standard measures) /fortified wine (glasses)/other alcoholic drinks (glasses) they consumed in an average week. Individuals reporting less than monthly drinking were asked how many measures of red wine (glasses)/white wine or champagne (glasses)/beer or cider(pints)/spirits(standard measures) /fortified wine (glasses)/other alcoholic drinks (glasses) they consumed in an average month. Each measure was assigned the value of 1 unit of alcohol apart from pints of beer and cider which were assigned 2 units. An average weekly intake was then calculated for each person. For monthly alcohol consumers, values were multiplied by 12 and then divided by 52 (**Supplementary Table 2**).

***Supplementary references***

1. Golding J, Pembrey M, Jones R, Team AS. ALSPAC--the Avon Longitudinal Study of Parents and Children. I. Study methodology. Paediatr Perinat Epidemiol. 2001;15(1):74-87.

2. Fraser A, Macdonald-Wallis C, Tilling K, Boyd A, Golding J, Davey Smith G, et al. Cohort Profile: the Avon Longitudinal Study of Parents and Children: ALSPAC mothers cohort. International journal of epidemiology. 2013;42(1):97-110.

3. ALSPAC. Data Dictionary <http://www.bris.ac.uk/alspac/researchers/data-access/data-dictionary/>; archived at <http://www.webcitation.org/6Tgld7Ze02014> [

4. Purcell S, Neale B, Todd-Brown K, Thomas L, Ferreira MA, Bender D, et al. PLINK: a tool set for whole-genome association and population-based linkage analyses. American journal of human genetics. 2007;81(3):559-75.

5. ALSPAC. GWAS Data Generation <http://www.bristol.ac.uk/alspac/researchers/resources-available/data-details/bio-resource/documents/gwas-data-generation.pdf?u07022013>: ALSPAC researchers; 2012 [

6. Rode L, Bojesen SE, Weischer M, Nordestgaard BG. High tobacco consumption is causally associated with increased all-cause mortality in a general population sample of 55,568 individuals, but not with short telomeres: a Mendelian randomization study. International journal of epidemiology. 2014;43(5):1473-83.

7. Holmen J, Midthjell K, Krüger Ø, Langhammer A, Holmen TL, Bratberg GH, et al. The Nord-Trøndelag Health Study 1995–97 (HUNT 2): objectives, contents, methods and participation. Norsk epidemiologi. 2003;13(1):19-32.

8. Krokstad S, Langhammer A, Hveem K, Holmen T, Midthjell K, Stene T, et al. Cohort profile: the HUNT study, Norway. International journal of epidemiology. 2013;42(4):968-77.

9. Bjørngaard J, Gunnell D, Elvestad M, Smith GD, Skorpen F, Krokan H, et al. The causal role of smoking in anxiety and depression: a Mendelian randomization analysis of the HUNT study. Psychological medicine. 2013;43(04):711-9.

10. Collins R. What makes UK Biobank special? Lancet. 2012;379(9822):1173-4.

11. Bycroft C, Freeman C, Petkova D, Band G, Elliott LT, Sharp K, et al. Genome-wide genetic data on~ 500,000 UK Biobank participants. bioRxiv. 2017:166298.

12. Mitchell R, Hemani G, Dudding T, Paternoster L. UK Biobank Genetic Data: MRC-IEU Quality Control, Version 1. 2017.

**Supplementary Table 1:** Derivation of units of alcohol per week in the ALSPAC, HUNT and CGPS cohorts.

|  | **ALSPAC** | **HUNT** | **CGPS** | **UK Biobank** |
| --- | --- | --- | --- | --- |
| **Question asked** | “How often have you drunk alcoholic drinks?” | “Concerning alcohol, are you a non-drinker?”  “How many glasses of beer/wine/spirits (separate questions for each) do you usually drink in the course of two weeks? (Do not include low-alcohol beer. Write 0 if less than once a month)” | “How often do you drink?” (Participants answered separately for beer, white wine, red wine, liqueur wines, liqueurs. | “In an average week/month, how many glasses of red wine/white wine or champagne/pints of beer or cider/spirits or liqueurs/fortified wine/other alcoholic drinks would you drink?” |
| **Answers given** | Never, less than 1 glass per week, 1+glasses per week, 1-2 glasses every day, at least 3-9 glasses every day, at least 10 glasses per day, where one glass means ½ pint of beer or cider, a small (125ml) glass of wine or a single pub measure (25ml) of spirit | Participants provided the number of glasses for each drink listed | Participants provided a number of ‘units per week’ for each drink listed above | Participants provided a number of ‘glasses per week/month’ (where beer and cider was specified as pints, wine was specified as a 125ml glass and spirits were specified as 2.8cl) for each drink listed about |
| **Derivation of variable** | Categories recoded as 0, 0.5, 3.5, 10.5, 42, 70 units per week | Addition of drinks per fortnight, divided by two to produce weekly consumption | Addition of units per week for each beverage to total weekly consumption | Beer was multiplied by 2 to obtain the number of units in a pint. Other drinks were counted as approx. 1 unit. For individuals who provided monthly values these were multiplied by 12 and divided by 52. Addition of units per week for each beverage to total weekly consumption. |
| **Mean (s.d.)** | 4.0 (6.1) | 3.0 (2.8) | 15.4 (15.8) | 13.7 (13.4) |

ALSPAC = Avon Longitudinal Study of Parents and Children; HUNT = Nord-Trøndelag Health Study; CGPS = Copenhagen General Population Study.

Non-drinkers were excluded across all datasets.

**Supplementary Table 2:** STROBE (Strengthening the Reporting of Observational Studies in Epidemiology) guidelines checklist.

|  | Item No | Recommendation | Completed |
| --- | --- | --- | --- |
| **Title and abstract** | 1 | (*a*) Indicate the study’s design with a commonly used term in the title or the abstract | ✓ |
|  |  | (*b*) Provide in the abstract an informative and balanced summary of what was done and what was found | ✓ |
| Introduction | | |  |
| Background/rationale | 2 | Explain the scientific background and rationale for the investigation being reported | ✓ |
| Objectives | 3 | State specific objectives, including any prespecified hypotheses | ✓ |
| Methods | | |  |
| Study design | 4 | Present key elements of study design early in the paper | ✓ |
| Setting | 5 | Describe the setting, locations, and relevant dates, including periods of recruitment, exposure, follow-up, and data collection | ✓ |
| Participants | 6 | (*a*) Give the eligibility criteria, and the sources and methods of selection of participants. Describe methods of follow-up | ✓ |
|  |  | (*b*) For matched studies, give matching criteria and number of exposed and unexposed | N/A |
| Variables | 7 | Clearly define all outcomes, exposures, predictors, potential confounders, and effect modifiers. Give diagnostic criteria, if applicable | ✓ |
| Data sources/ measurement | 8* | For each variable of interest, give sources of data and details of methods of assessment (measurement). Describe comparability of assessment methods if there is more than one group | ✓ |
| Bias | 9 | Describe any efforts to address potential sources of bias | ✓ |
| Study size | 10 | Explain how the study size was arrived at | ✓ |
| Quantitative variables | 11 | Explain how quantitative variables were handled in the analyses. If applicable, describe which groupings were chosen and why | ✓ |
| Statistical methods | 12 | (*a*) Describe all statistical methods, including those used to control for confounding | ✓ |
|  |  | (*b*) Describe any methods used to examine subgroups and interactions | ✓ |
|  |  | (*c*) Explain how missing data were addressed | ✓ |
|  |  | (*d*) If applicable, explain how loss to follow-up was addressed | N/A |
|  |  | (*e*) Describe any sensitivity analyses | ✓ |
| Results | | |  |
| Participants | 13* | (a) Report numbers of individuals at each stage of study—eg numbers potentially eligible, examined for eligibility, confirmed eligible, included in the study, completing follow-up, and analysed | ✓ |
|  |  | (b) Give reasons for non-participation at each stage | N/A |
|  |  | (c) Consider use of a flow diagram | ✓ |
| Descriptive data | 14* | (a) Give characteristics of study participants (eg demographic, clinical, social) and information on exposures and potential confounders | ✓ |
|  |  | (b) Indicate number of participants with missing data for each variable of interest | ✓ |
|  |  | (c) Summarise follow-up time (eg, average and total amount) | ✓ |
| Outcome data | 15* | Report numbers of outcome events or summary measures over time | ✓ |
| Main results | 16 | (*a*) Give unadjusted estimates and, if applicable, confounder-adjusted estimates and their precision (eg, 95% confidence interval). Make clear which confounders were adjusted for and why they were included | ✓ |
|  |  | (*b*) Report category boundaries when continuous variables were categorized | N/A |
|  |  | (*c*) If relevant, consider translating estimates of relative risk into absolute risk for a meaningful time period | N/A |
| Other analyses | 17 | Report other analyses done—eg analyses of subgroups and interactions, and sensitivity analyses | ✓ |
| Discussion | | |  |
| Key results | 18 | Summarise key results with reference to study objectives | ✓ |
| Limitations | 19 | Discuss limitations of the study, taking into account sources of potential bias or imprecision. Discuss both direction and magnitude of any potential bias | ✓ |
| Interpretation | 20 | Give a cautious overall interpretation of results considering objectives, limitations, multiplicity of analyses, results from similar studies, and other relevant evidence | ✓ |
| Generalisability | 21 | Discuss the generalisability (external validity) of the study results | ✓ |
| Other information | | |  |
| Funding | 22 | Give the source of funding and the role of the funders for the present study and, if applicable, for the original study on which the present article is based | ✓ |

**Supplementary Table 3.** Reporting of Mendelian randomization analysis based on guidelines in the IV reporting checklist and IV reporting flow chart

| **IV Reporting Checklist (Davies et al)** | | |
| --- | --- | --- |
| **#** | **Item** | **Reporting information** |
| 1 | State which population target parameter the study aims to estimate and the assumptions on which it depends | Methods: Statistical analysis |
| 2 | Report the association of instruments and exposure using a partial *F*-statistic | Supplementary tables 4 and 5 – R^2^ provided as this is more relevant to the analysis conducted here (see Methods: Statistical analysis) |
| 3 | Report and test the association of observed and potential confounding factors with both the exposure and the instrument | Supplementary Tables 4 and 5 |
| 4 | With multiple instruments report the test for overidentifying restrictions | N/A – multiple instruments not used |
| 5 | For binary outcomes, exposure and instruments, report a tabulation of the frequencies of each combination of instrument, exposure, and outcome, so readers can reconstruct basic results | N/A – outcome not binary |
| 6 | When using generalized linear models with binary outcomes, always use robust and bootstrapped standard errors and take clustering of study participants into account where necessary | N/A – outcome not binary |

Full Reference: Davies, Neil M., et al. "Issues in the reporting and conduct of instrumental variable studies: a systematic review." *Epidemiology* 24.3 (2013): 363-369.

**Supplementary Table 4:** Complete case demographics for analysis between smoking heaviness (cigarettes per day in individuals who smoke) and alcohol consumption (units per week) in ALSPAC by exposure and genotype.

|  | **Cigarettes per day prior to pregnancy (observational analysis)**  **N = 1359** | | | **Genotype (MR analysis, all available data)**  **Maximum N = 2198** | | | | | |
| --- | --- | --- | --- | --- | --- | --- | --- | --- | --- |
|  | **N**  **(**$\bar{\boldsymbol{x}}\boldsymbol{,}$ **s.d.)** | **Effect***  **(95% CI)** | **P** | **N** | **CC (%)** | **CT (%)** | **TT (%)** | **Effect***  **(95% CI)** | **P** |
| **Cigarettes per day prior to pregnancy** | - | - | - | 2198 | N = 979  $\bar{x}$ = 12.18, s.d. = 7.38 | N = 974  $\bar{x}$ = 13.20, s.d. = 7.78 | N = 245  $\bar{x}$ = 14.29, s.d. = 7.81 | coef = 1.04  (0.56 to 1.52) | ≤0.001 |
| **SEP**  - III manual, IV and V *(ref)*  - III non-manual, I and II | N = 349, $\bar{x}$ = 13.96, s.d. = 7.05  N = 1010, $\bar{x}$ = 12.09, s.d. = 7.75 | coef = -0.86  (-1.79 to 0.05) | 0.065 | 1593 | 193 (45.0)  517 (44.4) | 193 (45.0)  515 (44.2) | 43 (10.0)  132 (11.4) | OR = 1.04  (0.88 to 1.23) | 0.62 |
| **Age (years)** | N = 1359  $\bar{x}$ = 26.78 years, s.d. = 4.77 | coef = -0.03  (-0.12 to 0.05) | 0.47 | 2094 | N = 932  $\bar{x}$ = 26.39, s.d. = 4.93 | N = 930  $\bar{x}$ = 26.78, s.d. = 4.88 | N = 232  $\bar{x}$ = 26.58, s.d. = 4.96 | coef = 0.19  (-0.13 to 0.51) | 0.24 |
| **Partners smoking**  - No *(ref)*  - Yes | N = 587, $\bar{x}$ = 11.10, s.d. = 7.54  N = 772, $\bar{x}$ = 13.23, s.d. = 7.50 | coef = 2.14  (1.33 to 2.94) | ≤0.001 | 1921 | 352 (44.6)  487 (43.0) | 352 (44.6)  509 (45.0) | 85 (10.8)  136 (12.0) | OR = 1.07  (0.93 to 1.22) | 0.36 |
| **Partners drinking**  - No/very occasionally *(ref)*  - Yes | N = 405, $\bar{x}$ = 12.50, s.d. = 7.30  N = 954, $\bar{x}$ = 12.23, s.d. = 7.71 | coef = -0.26  (-1.14 to 0.62) | 0.56 | 2088 | 297 (44.9)  629 (44.1) | 290 (43.9)  638 (44.7) | 74 (11.2)  160 (11.2) | OR = 1.02  (0.88 to 1.17) | 0.78 |

R^2^ for association between rs1051730 and cigarettes per day prior to pregnancy = 0.008.

SEP = Socio-Economic Position.

*coefficients describe the unit increase in covariate for each additional cigarette smoked per day or each additional copy of the minor (T) allele.

**Supplementary Table 5:** Complete case demographics for analysis between smoking heaviness (cigarettes per day in individuals who smoke) and alcohol consumption (units per week) in UK Biobank by exposure and genotype.

|  | **Cigarettes per day prior to pregnancy (observational analysis)**  **N = 15 323** | | | **Genotype (MR analysis, all available data)**  **Maximum N = 15 462** | | | | | |
| --- | --- | --- | --- | --- | --- | --- | --- | --- | --- |
|  | **N**  **(**$\bar{\boldsymbol{x}}\boldsymbol{,}$ **s.d.)** | **Effect***  **(95% CI)** | **P** | **N** | **GG (%)** | **AG (%)** | **AA (%)** | **Effect***  **(95% CI)** | **P** |
| **Cigarettes per day prior to pregnancy** | - | - | - | 15 462 | N = 6784  $\bar{x}$ = 14.52, s.d. = 8.03 | N = 6979  $\bar{x}$ = 15.69, s.d. = 8.39 | N = 1699  $\bar{x}$ = 16.52, s.d. = 8.76 | coef = 1.06  (0.86 to 1.25) | ≤0.001 |
| **Education**  - Degree or higher *(ref)*  - A level or lower | N = 3025, $\bar{x}$ = 13.47, s.d. = 8.14  N = 12 298, $\bar{x}$ = 15.70, s.d. = 8.30 | OR = 2.23  (1.90 to 2.56) | ≤0.001 | 15 323 | 1303 (19.38)  5422 (80.62) | 1394 (20.17)  5518 (79.83) | 328 (19.45)  1358 (80.55) | OR = 0.98  (0.92 to 1.04) | 0.545 |
| **Age (years)** | N = 15 323, $\bar{x}$ = 54.63, s.d. = 8.08 | coef = 0.01  (-0.01 to 0.03) | 0.094 | 15 462 | N = 6784  $\bar{x}$ = 54.74, s.d. = 8.09 | N = 6979  $\bar{x}$ = 54.61, s.d. = 8.09 | N = 1699  $\bar{x}$ = 54.46, s.d. = 8.03 | coef = -0.14  (-0.33 to 0.06) | 0.166 |
| **Sex**  - Male *(ref)*  - Female | N = 8204, $\bar{x}$ = 16.86, s.d. = 8.87  N = 7119, $\bar{x}$ = 13.42, s.d. = 7.20 | OR = -3.44  (-3.70 to -3.18) | ≤0.001 | 15 462 | 3612 (53.24)  3172 (46.72) | 3770 (54.02)  3209 (45.98) | 903 (53.15)  796 (46.85) | OR = 0.99  (0.95 to 1.04) | 0.703 |

R^2^ for association between rs16969968 and cigarettes per day prior to pregnancy = 0.0479.

*coefficients describe the unit increase in covariate for each additional cigarette smoked per day or each additional copy of the minor (A) allele.

**Supplementary Table 6:** Association between rs1051730/rs16969968 and alcohol consumption by smoking status in ALSPAC and UK Biobank sample (MR analysis).

| **Dataset** | **Smoking Status** | **All available Data** | | | | **Complete Case Analysis** | | | |
| --- | --- | --- | --- | --- | --- | --- | --- | --- | --- |
|  |  | **N** | **Effect***  **(95% CI)** | **LR(χ^2^)** | **LR test P value** | **N** | **Effect***  **(95% CI)** | **LR(χ^2^)** | **LR test P value** |
| **ALSPAC**  **(rs1051730)** | Current | 2250 | coef = -0.18  (-0.69 to 0.34) | 0.46 | 0.50 | 1359 | coef = -0.12  (-0.72 to 0.48) | 0.15 | 0.70 |
|  | Former | 1337 | coef = -0.16  (-0.30 to 0.61) | 0.46 | 0.50 | 1017 | coef = 0.23  (-0.28 to 0.74) | 0.75 | 0.39 |
|  | Never | 3801 | coef = 0.04  (-0.15 to 0.22) | 0.18 | 0.67 | 2924 | coef = -0.01  (-0.21 to 0.19) | 0.01 | 0.91 |
| **UK Biobank**  **(rs16969968)** | Current | 24 372 | coef = -0.01  (-0.03 to 0.01) | 0.64 | 0.43 | 24 155 | coef = -0.01  (-0.03 to 0.01) | 0.65 | 0.42 |
|  | Former | 95 731 | coef = -0.00  (-0.01 to 0.01) | 0.15 | 0.63 | 94 984 | coef = 0.01  (-0.01 to 0.01) | 0.14 | 0.63 |
|  | Never | 136 230 | coef = -0.00  (-0.01 to 0.00) | 1.68 | 0.27 | 135 256 | coef = -0.01  (-0.01 to 0.01) | 2.10 | 0.21 |

*coefficients describe the increase in units of alcohol per week for each additional copy of the minor (T) allele

LR = Likelihood ratio

All results have been adjusted for age

**Supplementary Figure 1:** Meta-analysis of the associations between genetic variant and units of alcohol per week for the ALSPAC, CGPS, HUNT and UK Biobank cohorts, in ever and never smokers.


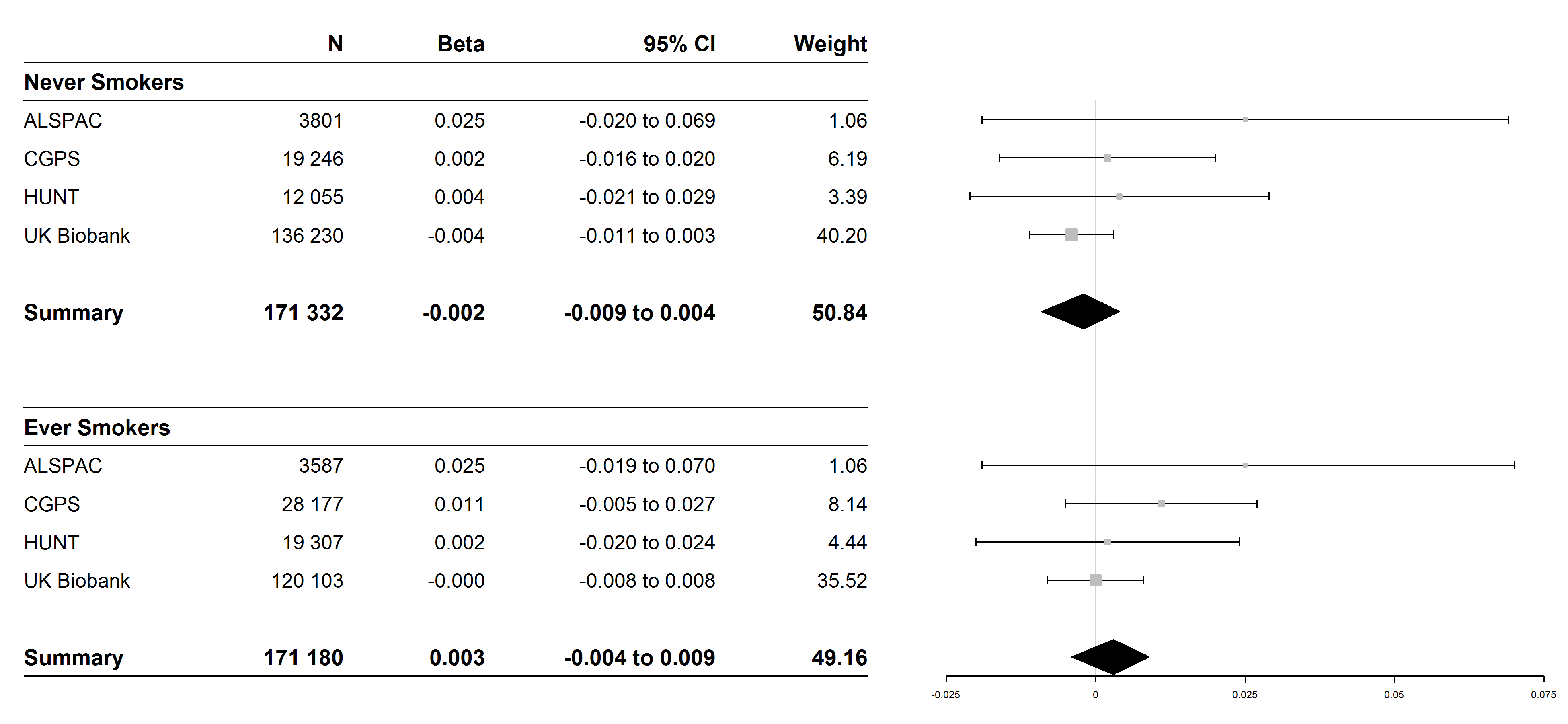


Effect sizes represent the standard deviation increase in units of alcohol per week for each additional copy of the minor (risk) allele.

P values for association in: never smokers = 0.500; ever smokers = 0.448.

Test of heterogeneity: never smokers I^2^ = 0.0%, p = 0.537; ever smokers I^2^ = 0.0%, p = 0.456.

Note: Weights are from random effects analysis.

**Supplementary Figure 2:** Meta-analysis of the associations between genetic variant and units of alcohol per week for the ALSPAC, CGPS, HUNT in never, former and current smokers. Associations in UK Biobank have been removed in case of collider bias.


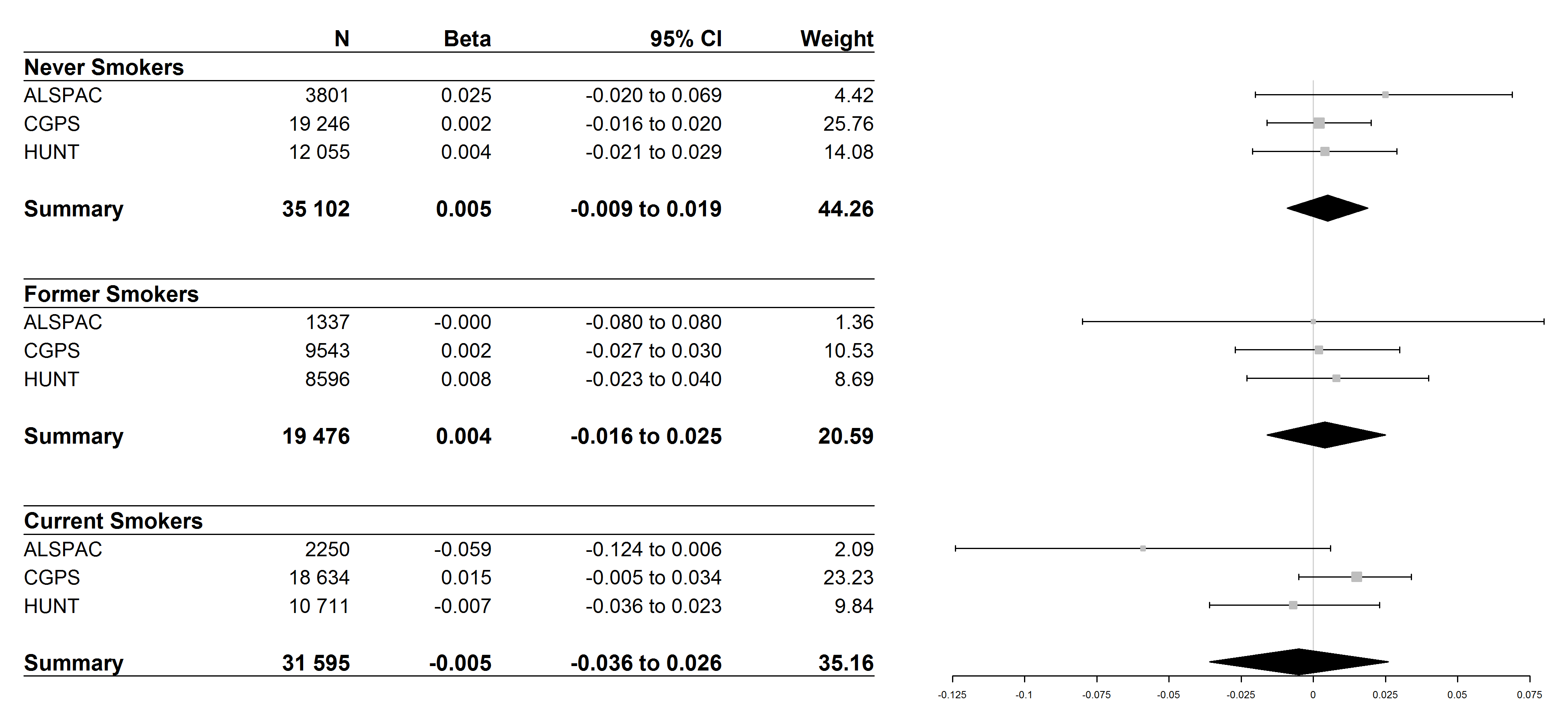


Effect sizes represent the standard deviation increase in units of alcohol per week for each additional copy of the minor (risk) allele.

P values for association in: never smokers = 0.505; former smokers = 0.680; current smokers: 0.736.

Test of heterogeneity: never smokers I^2^ = 0.0%, p = 0.651; former smokers I^2^ = 0.0%, p = 0.948, current smokers I^2^ = 64.2%, p=0.070.

Note: Weights are from random effects analysis.
